# The Development of Dual Vaccines against Lumpy Skin Disease (LSD) and Bovine Ephemeral Fever (BEF)

**DOI:** 10.3390/vaccines9111215

**Published:** 2021-10-20

**Authors:** Nicola Douglass, Ruzaiq Omar, Henry Munyanduki, Akiko Suzuki, Warren de Moor, Paidamwoyo Mutowembwa, Alri Pretorius, Tshifhiwa Nefefe, Antoinette van Schalkwyk, Pravesh Kara, Livio Heath, Anna-Lise Williamson

**Affiliations:** 1 Division of Medical Virology, Department of Pathology, Faculty of Health Sciences, University of Cape Town, Cape Town 7925, South Africa; omar.ruzaiq@gmail.com (R.O.); Henry.munyanduki@pirbright.ac.uk (H.M.); SZKAKI001@myuct.ac.za (A.S.); warren.demoor@uct.ac.za (W.d.M.); Anna-Lise.Williamson@uct.ac.za (A.-L.W.); 2 Institute of Infectious Disease and Molecular Medicine, University of Cape Town, Cape Town 7925, South Africa; 3 Onderstepoort Veterinary Institute, ARC, Pretoria 0110, South Africa; mutowembwap@arc.agric.za (P.M.); PretoriusAL@arc.agric.za (A.P.); nefefet@arc.agric.za (T.N.); VanSchalkwykA1@arc.agric.za (A.v.S.); KaraP@arc.agric.za (P.K.); HeathL@arc.agric.za (L.H.)

**Keywords:** lumpy skin disease virus, bovine ephemeral fever virus, dual vaccine, neutralization, LSDV challenge

## Abstract

Dual vaccines (*n* = 6) against both lumpy skin disease (LSD) and bovine ephemeral fever (BEF) were constructed, based on the BEFV glycoprotein (G) gene, with or without the BEFV matrix (M) protein gene, inserted into one of two different LSDV backbones, nLSDV∆SOD-UCT or nLSDVSODis-UCT. The inserted gene cassettes were confirmed by PCR; and BEFV protein was shown to be expressed by immunofluorescence. The candidate dual vaccines were initially tested in a rabbit model; neutralization assays using the South African BEFV vaccine (B-Phemeral) strain showed an African consensus G protein gene (Gb) to give superior neutralization compared to the Australian (Ga) gene. The two LSDV backbones expressing both Gb and M BEFV genes were tested in cattle and shown to elicit neutralizing responses to LSDV as well as BEFV after two inoculations 4 weeks apart. The vaccines were safe in cattle and all vaccinated animals were protected against virulent LSDV challenge, unlike a group of control naïve animals, which developed clinical LSD. Both neutralizing and T cell responses to LSDV were stimulated upon challenge. After two inoculations, all vaccinated animals produced BEFV neutralizing antibodies ≥ 1/20, which is considered protective for BEF.

## 1. Introduction

Lumpy skin disease (LSD) and bovine ephemeral fever (BEF) are two cattle diseases of economic importance with low mortality but high morbidity rates [1,2,3]. LSD is classified as a notifiable disease by the World Organization for Animal Health (OIE); it is characterized by fever, clinical lesions which affect animal hides, reduced milk yields and abortion in pregnant ewes [2,4]. It was initially confined to Africa, but spread to Egypt in 1988, Israel in 1989, followed by the Middle East in the 1990s [5]. More recently, it has spread to Europe and Asia [3,4,6,7]

BEF is caused by an RNA virus, belonging to the family *Rhabdoviridae*, genus *Ephemerovirus,* group *Lyssavirus*. Clinically, it presents as a transient disease of 3–4 days, causing sudden fever, salivation, nasal discharge, stiffness and can cause reduced milk production as well as abortion in pregnant ewes and infertility in bulls [1,8]. The disease is endemic in Africa, the Middle East, Asia and Australia [9,10,11]. A live attenuated vaccine, B-phemeral is commercially available as a two-dose vaccine regimen, supplied by Onderstepoort Biological Products (OBP) [12].

**Figure 1:**
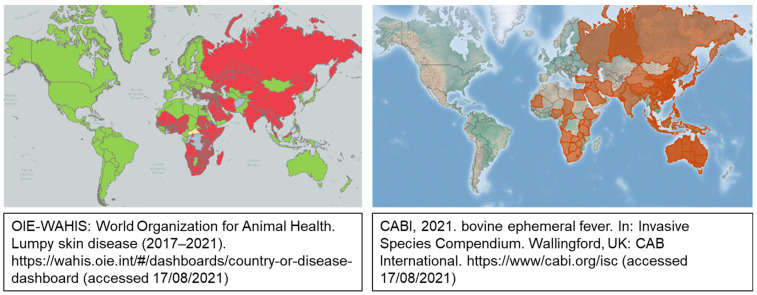
Geographical distribution of LSDV and BEFV. LSDV information was obtained from the OIE-WAHIS World Organization for Animal Health. LSD disease status is represented as: red—present, yellow—suspected, green—absent, grey—no information available. BEFV information was obtained from CABI.

Geographically, BEF and LSD show considerable overlap, notably in Africa (Figure 1). Both diseases are seasonal, with the causative viruses being spread by biting insects. Ideally, vector control could reduce the incidence of both viral infections, but the implementation of this intervention is impractical. Effective vaccines are available for LSD and BEF, but, due to the seasonal nature of the diseases, are not always considered necessary by animal owners. The development of a single vaccine for control of the two viral diseases would be attractive to both cattle owners as well as vaccine manufactures, due to the reduction in cost and number of vaccines administered. In addition, concomitant vaccination against the two diseases will automatically reduce the incidence of BEF, which is largely under-reported, despite being of great economic importance.

Bovine ephemeral fever virus (BEFV) has a single serotype worldwide and, although the presently available vaccines protect against all strains, it is known that neutralising antibodies appear higher against homologous strains [11]. The BEFV glycoprotein (G) has been shown to be immunogenic both as a protein [13] and recombinant vaccinia virus vaccine [14]. The protein has been well characterized immunologically and four neutralizing epitopes have been identified [15,16,17]. In this study, separate candidate vaccines were made, based either on the Walker G protein sequence, referred to as Ga, or a consensus South African G protein sequence, referred to as Gb, derived from published South African sequences [18]. In addition, two vaccines were designed to express the matrix (M) protein gene together with Gb. The BEFV matrix protein gene is highly conserved and the Walker and South African M protein sequences are identical [12,19].

Poxviruses have been recognized as excellent vectors for vaccines against human and animal pathogens, eliciting both humoral and cellular protective immune responses [20]. Lumpy skin disease virus (LSDV), a member of the *Poxviridae* family, genus *Capripoxviridae*, is both the causative agent of lumpy skin disease (LSD) as well as an attractive vaccine vector for cattle. With the ability to accommodate large insertions, it can be engineered as a mono- or multi-valent vaccine [21,22,23,24]. Multiple safe and effective LSDV vaccines are available and five have been compared with respect to virological, clinical and serological properties [25]. In particular, the live attenuated Neethling vaccine strain, made by Onderstepoort Biological Products (OBP), has been shown to be highly effective in protecting cattle against LSD [26,27]. Recently, a killed version of this vaccine has been shown to protect against virulent LSDV challenge [28].

Our group has made two variants of the OBP Neethling vaccine strain, nLSDV∆SOD-UCT and nLSDVSODis-UCT; these correspond to superoxide dismutase (SOD) gene homologue knock-out and knock-in mutants, respectively [29]. nLSDVSODis-UCT has a full-length SOD homologue gene which was stabilized by the alteration of nucleotide sequences in a run of AT binucleotides such that the amino acid sequence of the full-length SOD homologue was retained [30]. Both nLSDV∆SOD-UCT and nLSDVSODis-UCT were used as vector backbones to express one or two bovine ephemeral fever virus (BEFV) genes, with the aim of generating dual vaccines against LSDV and bovine ephemeral fever virus (BEFV). In total, six candidate LSDV-BEFV vaccines were constructed. These were first tested in a rabbit model for neutralization responses to BEFV and LSDV, and thereafter, two candidate vaccines were selected for testing in cattle, for immunogenicity against LSDV and BEFV; and challenge against virulent LSDV. The aim was to identify a dual vaccine which would induce neutralizing responses to both BEFV and LSDV. In addition, LSDV- and BEFV-specific T cell responses would be desirable. The ultimate goal was to obtain protection against virulent LSDV challenge.

## 2. Materials and Methods

### 2.1. Viruses and Vaccine Preparation

#### 2.1.1. LSDV

Modified LSDV Neethling vaccines, nLSDV∆SOD-UCT and nLSDVSODis-UCT, were available within our group [29] and used as vector backbones in the construction of dual vaccines against LSDV and BEFV. Candidate vaccines tested in rabbits were grown in Madin Darby bovine kidney (MDBK) cells (CCL-22), obtained from ATCC, and infected at a multiplicity of infections (MOI) of 0.2 for 6 days. Because these cells harbour bovine viral diarrhea virus (BVDV), vaccines used for the cattle experiment were passaged twice through specific pathogen free (SPF) eggs from White Leghorn chickens (AviFarms, South Africa) [31] to remove BVDV, before being grown to higher titres in primary lamb testes cells, which were prepared from foetal lamb testes (LT) obtained through the animal unit at the University of Cape Town, and infected at an MOI of 0.001 for 7 days. Infected cells were freeze/thawed three times and, after a clarifying centrifugation step, the virus was pelleted through a 36% sucrose cushion, resuspended in phosphate-buffered saline (PBS) (Gibco, Waltham, MA, USA), aliquoted and stored at −80 °C. Vaccines were titrated in MDBK cells using the TCID_50_ method of Reed and Muench [32]. All recombinant vaccines were confirmed to be correct by PCR amplification across the LSDV49-50 loci (Section 2.3), followed by Sanger sequencing of the amplified products (done by the Central Analytical Facility at Stellenbosch University). BEFV gene expression was shown by immunofluorescence (Section 2.4).

#### 2.1.2. BEFV

BEFV vaccine B-Phemeral (OBP, Pretoria, South Africa) was used for neutralization assays. The virus was grown in baby hamster kidney (BHK-21) cells (CCL-10), obtained from ATCC, and infected at an MOI of 0.2 for 4–5 days. Concentrated virus was resuspended in PBS, aliquoted and stored at −80 °C. The virus was titrated in BHK-21 cells using the TCID_50_ method of Reed and Muench [32].

### 2.2. Construction of Recombinant LSDV-BEFV Vaccine Candidates

The components of the different vaccines made are described in Section 2.2.1 and have been summarized in Table 1.

#### 2.2.1. Transfer Vector Design

Transfer vectors were designed such that the foreign gene cassettes were placed between flanking sequences corresponding to the ends of convergent LSDV open reading frames (ORFs) 49 (positions 1682 to 2031) and 50 (positions 1 to 450) from LSDV sequence AF409138.1 obtained from GenBank [37].

The Australian Walker BEFV G protein gene was obtained from GenBank: M94266.1 [14] and was placed under the control of the modified vaccinia virus promoter (PmH5) (GenBank: FJ386852.1) [33]. The Australian G protein gene (Ga) was inserted together with the mCherry fluorescent marker gene under the control of a fowlpox virus promoter (PmFPV), which was identified as a bidirectional promoter [34], but was modified such that the early promoter was retained and the late promoter removed [38]. The transfer vector pBEFV_Ga_mCherry, containing the elements required for homologous recombination with nLSDV∆SOD-UCT and nLSDVSODis-UCT, was used to generate LSDV(∆SOD)BEFV-Ga and LSDV(SODis)BEFV-Ga, respectively.

The South African BEFV G gene, Gb, was derived from a consensus sequence of 14 BEFV isolates collected between 1968 and 1999 [18] and placed under the control of PmH5 (GenBank: FJ386852.1) [33]. This gene was inserted together with a green fluorescent protein gene, eGFP, under the control of the synthetic vaccinia virus promoter (pS) [35,36] into nLSDV∆SOD-UCT and nLSDVSODis-UCT, using transfer vector pBEFV_Gb_eGFP, to generate recombinant viruses LSDV(∆SOD)BEFV-Gb and LSDV(SODis)BEFV-Gb, respectively.

LSDV(∆SOD)BEFV-Gb-M and LSDV(SODis)BEFV-Gb-M were constructed using transfer vector pBEFV_Gb_M_eGFP, which was equivalent to pBEFV_Gb_eGFP, but, in addition, had the matrix protein gene, M, under the control of PmFPV, downstream of the Gb gene.

All the elements described above were commercially synthesized by GenScript (Hong Kong). The amino acid sequences of the genes described were retained; however, nucleotide sequences were modified to remove runs of four or more Cs and Gs, poxvirus transcription termination sites (T5NT) and unwanted restriction enzyme sites.

#### 2.2.2. Construction of LSDV(∆SOD)BEFV-Ga, LSDV(∆SOD)BEFV-Gb and LSDV(∆SOD)BEFV-Gb-M

The three recombinants, LSDV(∆SOD)BEFV-Ga, LSDV(∆SOD)BEFV-Gb and LSDV(∆SOD)BEFV-Gb-M, were isolated in the following way: primary foetal lamb testes (LT) cells were infected with the parental virus, nLSDV∆SOD-UCT (at MOI of 0.25 for construction of LSDV(∆SOD)BEFV-Ga and MOI of 0.01 for the other two recombinants) whilst still in suspension after trypsinization, and plated in 12-well plates at 5 × 10^5^ cells per well. Twenty-four hours post infection, cells were transfected with transfer vectors pBEFV_Ga_mCherry, pBEFV_Gb_eGFP and pBEFV_Gb_M_eGFP, respectively, using X-tremeGene HP (Roche, Basel, Switzerland) according to the manufacturer’s instructions. Infected and transfected cells were incubated for a further 2 (for LSDV(∆SOD)BEFV-Ga and LSDV(∆SOD)BEFV-Gb-M) or 3 (for LSDV(∆SOD)BEFV-Gb) days. Crude lysates were recovered by freezing and thawing the cells twice and passaged on MDBK cells. Fluorescing foci were picked 3–5 days post infection and suspended in Dulbecco’s Modified Eagle Medium (DMEM) GlutaMax with high glucose (Gibco, Waltham, MA, USA). The picked foci were lysed by two rounds of freeze/thawing and the lysate used to infect fresh MDBK cells. This procedure was repeated seven to thirteen times per recombinant, until only fluorescent foci were visible, at which stage the recombinants were confirmed to be correct by PCR. Before high titre stocks were prepared (as described in Section 2.1.1), the recombinant viruses were diluted and passaged in 96-well plates such that, for each recombinant, a single focus in a single well was purified and expanded.

#### 2.2.3. Construction of LSDV(SODis)BEFV-Ga, LSDV(SODis)BEFV-Gb and LSDV(SODis)BEFV-Gb-M

Primary LT cells were seeded into 12-well plates 18 h prior to infection, such that they were approximately 80% confluent, in a logarithmic phase of growth. Monolayers were infected with nLSDVSODis-UCT, at an MOI of 1, for 2 h before being transfected with the appropriate transfer vector (pBEFV_Ga_mCherry for LSDV(SODis)BEFV-Ga, pBEFV_Gb_eGFP for LSDV(SODis)BEFV-Gb and pBEFV_Gb_M_eGFP for LSDV(SODis)BEFV-Gb-M), using X-tremeGene HP (Roche, Basel, Switzerland). Three days post transfection, the cells were freeze/thawed and recombinant fluorescing foci were purified in MDBK cells as described in Section 2.2.2. Using this method, the parental virus was lost earlier, by passage 4, after which single foci were purified from single wells of 96-well plates using end-point dilutions.

### 2.3. PCR Confirmation of LSDV-BEFV Recombinants

The insertion of the foreign gene cassette between LSDV ORFs 49 and 50 was confirmed by polymerase chain reaction (PCR) followed by agarose gel electrophoresis and Sanger DNA sequencing of the amplicon. The primer sequences used were 5′-GAGTGAAGCCTGGAACAT-3′ (forward) and 5′-ACTCTATCGCATCTGGAAACT-3′ (reverse). These generated fragment sizes of 1329 bp for the parent viruses nLSDV∆SOD-UCT and nLSDVSODis-UCT, 4816 bp for LSDV(∆SOD)BEFV-Ga and LSDV(SODis)BEFV-Ga, 4842 bp for LSDV(∆SOD)BEFV-Gb and LSDV(SODis)BEFV-Gb and 5574 bp for LSDV(∆SOD)BEFV-Gb-M and LSDV(SODis)BEFV-Gb-M. Phusion High-Fidelity enzyme was used with HF Buffer (New England BioLabs, Ipswich, MA, USA). The following thermocycling parameters were used for all PCR reactions: initial denaturation at 98 °C for 5 min followed by 40 cycles of denaturation at 98 °C for 30 s, annealing at 56 °C for 30 s, extension at 72 °C for 6 min and final extension at 72 °C for 10 min. PCR products were separated on 0.8% agarose gels, containing 0.5 μg/mL ethidium-bromide, by electrophoresis in 1× TBE buffer.

### 2.4. Immunofluorescence

Rabbit antiserum to B-phemeral virus was obtained from Stellenbosch University. A rabbit was immunized intravenously into the marginal ear vein with nine inoculations of B-phemeral virus administered alone (days 0, 3, 10, 17, 29, 38, 55, 58, 65), followed by nine inoculations of BEFV complexed with naked bacteria, six weeks later (days 0, 4, 7, 15, 18, 22, 28, 32 and 35) [39]. The final bleed was taken on day 42 of the second set of inoculations.

Immunofluorescence was used to detect BEFV protein in cells infected with the six recombinants, LSDV(∆SOD)BEFV-Ga, LSDV(SODis)BEFV-Ga, LSDV(∆SOD)BEFV-Gb, LSDV(SODis)BEFV-Gb, LSDV(∆SOD)BEFV-Gb-M and LSDV(SODis)BEFV-Gb-M. As controls, cells were infected with nLSDV∆SOD-UCT or nLSDVSODis-UCT (negative controls) or B-phemeral (positive control). MDBK cells, seeded in chamber slides, were infected at an MOI of 0.1 for 48 h. The cells were washed and fixed with 4% paraformaldehyde for 10 min, followed by methanol for 30 s before being washed twice with PBS for 10 s. The slides were incubated overnight with rabbit serum (diluted 1:1000), which had been pre-adsorbed with the lysed cellular debris of approximately 4 × 10^7^ MDBK cells, which were freeze/thawed and centrifuged at 15,000× *g* for 10 min. The rabbit serum was added to the cellular debris in 10 mL PBS with 2% BSA and pre-adsorbed for 4 h, after which the cell debris was removed by centrifugation at 3000× *g* for 7 min and the supernatant was used. After incubation with primary antibody the cells were washed twice with PBS and the secondary antibody was added. For detection of BEFV protein from recombinants expressing Ga, cells were treated with donkey anti-rabbit Alexa488 (green) secondary antibody (Sigma) (diluted 1:500); for detection of BEFV expression from recombinants expressing Gb and Gb-M, cells were treated with donkey anti-rabbit CY3 (red) secondary antibody (Sigma) (diluted 1:500). Cells were incubated with secondary antibody for 1.5 h, washed with PBS (2 × 10 min) and stained with Hoechst solution (1 uL Hoechst in 5 mL PBS) for 1 min. After two washes with PBS (10 min each), the top of the chamber slide was removed and the slide allowed to air dry for 5 min. A drop of mowiol with n-propylgallate (anti-fade) was added and a coverslip placed over the cells. The slides were viewed the following day using an inverted Zeiss LMS 880 with airyscan confocal microscope (Zeiss, Oberkochen, Germany).

### 2.5. Rabbit Immunization

All candidate LSDV-BEFV vaccines were tested in female New Zealand white rabbits of approximately 2 months old, weighing > 2 kg each, with five animals per group. One rabbit died during the acclimatization period and so the control group (inoculated with nLSDV∆SOD-UCT) had only four rabbits. The vaccines were administered intramuscularly (i.m.) as two inoculations of 500 uL into each hind leg. Each animal received three homologous doses of 10^6^ TCID_50_ given at four-week intervals. Two weeks after the final inoculation blood was collected by cardiac puncture. Serum was heat-inactivated at 56 °C for 45 min.

The rabbit experiments were performed at Stellenbosch University in an insect-free facility and animals were handled by an experienced veterinary surgeon and animal technicians. Approval to perform these experiments was granted by the animal ethics committee at the University of Cape Town, FHS reference number 018_039 and South African Department of Agriculture, Forestry and Fisheries reference number 12/11/1/7/1.

### 2.6. Cattle Immunization and Challenge with Virulent LSDV

The two candidate vaccines LSDV(∆SOD)BEFV-Gb-M and LSDV(SODis)BEFV-Gb-M were tested in Friesian cattle at the ARC—Onderstepoort Veterinary Research Institute (Transboundary Animal Diseases) facility. Permission was granted to do this experiment by the South African Department of Agriculture, Land Reform and Rural Development (DALRRD), reference number 12/11/1/1. Two groups (*n* = 10) of cattle > 6 months of age, shown to be LSDV negative by the serum neutralization test (SNT), were vaccinated subcutaneously with LSDV(∆SOD)BEFV-Gb-M and LSDV(SODis)BEFV-Gb-M, respectively, with an initial inoculation (day 0) of 10^5^ TCID_50_ per animal followed by a homologous boost of 5 × 10^4^ TCID_50_ on day 32. Serum was prepared from blood samples collected at days 0, 14 and 28 post vaccination (dpv); and 14, 30 and 169 days post boost (dpb) and inactivated at 60 °C for 30 min.

On day 201 (169 dpb), cattle were challenged with a total of 1 × 10^7^ TCID_50_ virulent LSDV (LSDV/Cradock-EC/RSA/1958) per animal, administered intradermally at multiple sites (to mimic vector biting) and intravenously in a 2 mL volume in the neck vein. A control group of three naïve animals, shown to be LSDV seronegative by SNT, were also inoculated in the same way. All animals were monitored for 28 days post challenge and rectal temperatures were recorded. Blood was taken on days 0, 7, 14, 21 and 28 days post challenge (dpc) for whole blood T cell assays as well as neutralization assays (LSDV and BEFV) and BEFV Enzyme-Linked Immunosorbent Assay (ELISA).

### 2.7. LSDV Neutralization

Serum samples were sent to the Diagnostics Services Programme laboratory of the ARC-Onderstepoort Veterinary Institute for lumpy skin disease serum neutralization (LDV-SNT) testing. A neutralization titre greater than or equal to 1/4 was considered positive.

### 2.8. BEFV Neutralization

An in-house BEFV neutralization test was set up at the University of Cape Town to test the ability of serum to neutralize infection of BHK-21 cells with BEFV (B-phemeral vaccine (OBP)). Two-fold serial dilutions were made of sera, starting with 1/5, in DMEM (Gibco, Waltham, MA, USA) supplemented with 1% penicillin-streptomycin (Gibco, Waltham, MA, USA). BEFV was diluted in 1 × PBS to obtain 50 pfu/50 μL (rabbit experiments) or 25 pfu/50 μL (cattle experiment). To each well of a 96-well tissue culture plate, 50 μL of each serial dilution of serum and 50 μL of the diluted BEFV were added. Each serum dilution was tested in ten replicates for the sera from the vaccinated animals and in eight replicates for the pre-immune sera. Following a 2-h incubation at 37 °C in a CO_2_ incubator, 100 μL of BHK-21 cells at a concentration of 2 × 10^5^ cells/well were seeded and plates were further incubated at 37 °C for 4 days. Wells containing BHK-21 cells only and wells with BEFV + BHK-21 cells were included in each plate as a negative control and virus control, respectively. For the testing of the cattle serum, a rabbit serum sample was selected as an internal positive control. Neutralization titres were determined as the reciprocal of the highest dilution of serum showing inhibition of BEFV infection in ≥50% of BEFV-infected wells [40]. Final SNTs were taken from experiments which showed reproducibility of the positive control. A neutralization titre greater than or equal to 1/5 was considered positive.

### 2.9. BEFV Enzyme-Linked Immunosorbent Assay (ELISA)

The presence of binding antibodies to BEFV was tested using a BEFV antibody ELISA kit (Unibiotest, Wuhan, China). Pre-immune sera and sera from 14 dpb and 30 dpb were diluted 1:10 and added to each well of the 96-well microtiter plates provided, which had been coated with BEFV G protein antigen derived from a truncated Asian sequence. Following 30-min to 1-h incubation at 37 °C, the plates were washed thrice with 1 × PBS containing 0.1% Tween 20 (PBS-T), and 100 μL of rabbit-anti-bovine IgG antibody conjugated with horseradish peroxidase (HRP) were added to each well. The plates were incubated for 30 min to 1 h at 37 °C. After the plates were washed thrice with PBS-T as described previously, 100 μL of 3,3′,5,5′-tetramethylbenzidine (TMB) substrate was added to each well, and the plates were incubated in dark for 10 to 15 min at 37 °C. The enzymatic reaction was terminated by the addition of 100 μL stop solution to each well. Absorbance was measured at 450 nm (OD_450_) using a Versa Max Microplate reader and SoftMax Pro Software version 6.3 (Molecular Devices, San Jose, CA, USA). Positive and negative controls were included in the ELISA kit and each sample was tested in duplicate or triplicate. Sera with OD_450_ ≤ 0.22 were regarded as negative and OD_450_ > 0.30 as positive.

### 2.10. T Cell Assays

Samples were collected pre-challenge and 7, 14 and 21 days post challenge (dpc). Whole blood was mixed 1:1 with medium [RPMI (Gibco, Waltham, MA, USA) plus 1% penicillin-streptomycin (Sigma-Aldrich, St Louis, MO, USA)] referred to as the unstimulated control or with LSDV Neethling strain, BEFV virus or with BHK-21 cells at a 1:10 dilution in medium. Blood was seeded in 96-well plates in triplicate with a total volume of 200 µL/well. Blood was incubated for 24 h in a humidified, 5% CO_2_ incubator at 37 °C and brefeldin A was added during the last 5 h of incubation. Red blood cells were removed by lysis using BD Pharm Lyse™ lysing solution (BD Biosciences, Franklin Lakes, NJ, USA), cells were fixed with 4% paraformaldehyde, perforated (0.05% Saponin) and then stained with anti-CD4-FITC (1:50 dilution; BioRad, Hercules, CA, USA); anti-CD8-PE (BioRad, 1:20 dilution) and Mouse anti-Bovine Interferon Gamma:Alexa Fluor^®^647 (1:100 dilution; BioRad, Hercules, CA, USA). Samples were assayed on a FC 500 Beckman Coulter flow cytometer and data analysed using Kaluza version 2.1 (Beckman Coulter, Brea, CA, USA). Values significantly higher than unstimulated control (*p* ≤ 0.05) were considered positive. The 21 dpc analyses data only included anti-CD8 antibodies and IFNγ due to a shortage of anti-CD4 antibody.

### 2.11. Statistical Analysis

Statistical analysis was performed using GraphPad Prism 8 (GraphPad Software, San Diego, CA, USA). A parametric *t*-test or non-parametric Mann–Whitney U test were used for the comparison between two different vaccine groups. Analysis for the unpaired multiple comparison between different time points within a single vaccine group or different vaccine groups at a single time point was performed using Welch one-way analysis of variance (ANOVA) with Dunnett’s T3 multiple comparison test or Kruskal–Wallis test with Dunn’s multiple comparison test. The paired multiple comparison between different time points within a single vaccine group was performed using repeated measures ANOVA test with Tukey’s multiple comparison test. *p*-values less than 0.05 were considered statistically significant.

### 2.12. Ethics

Authorization to grow LSDV in eggs was granted by the University of Cape Town Animal Ethics committee, (018/012). Ethics approval to test the candidate vaccines in rabbits was granted from the University of Cape Town (AEC 018_039), Stellenbosch University (UCT-DOUG-2019) and the South African Department of Agriculture, Forestry and Fisheries (DAFF), ref: 12/11/1/7/1. Ethics approval for the cattle experiment to be performed at the ARC institute—Onderstepoort Veterinary Research (Transboundary Animal Diseases) was granted by the South African Department of Agriculture, Land Reform and Rural Development (DALRRD), study number: TADP-S-20/02, [DALRRD Ref no: 12/11/1/1].

## 3. Results

### 3.1. Construction and Confirmation of Candidate Vaccines against LSDV and BEFV

**Figure 2:**
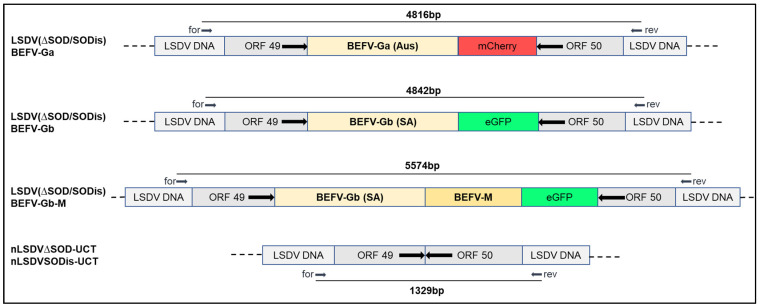
Diagrammatical representation of six dual LSDV-BEFV candidate vaccines constructed. The foreign gene cassettes were inserted into either nLSDV∆SOD-UCT or nLSDVSODis-UCT, between LSDV ORFs 49 and 50. A BEFV G protein sequence was derived from an Australian isolate (Ga) or a South African consensus sequence (Gb). M = Matrix protein gene; mCherry and eGFP encode red and green fluorescent marker proteins, respectively. The positions of primer binding sites (for = forward and rev = reverse) are shown, as well as the PCR product sizes amplified from these primers.

**Figure 3:**
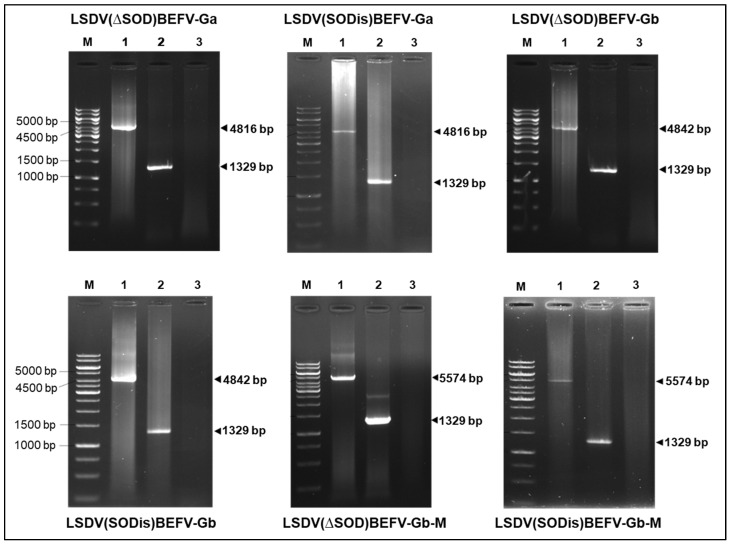
PCR confirmation of the recombinant LSDV-BEFV candidate vaccines. DNA was extracted from infected MDBK cells and subjected to PCR using forward (for) and reverse (rev) primers as indicated in Figure 2. Fragments were separated by electrophoresis on 0.8% agarose gels. M—GeneRuler 1 kb DNA Ladder (Thermo Fisher Scientific), lanes 1 = vaccine of interest, as labelled above or below the images; lanes 2 = parent LSDV (nLSDV∆SOD-UCT or nLSDVSODis-UCT) and lanes 3 = uninfected cells.

**Figure 4:**
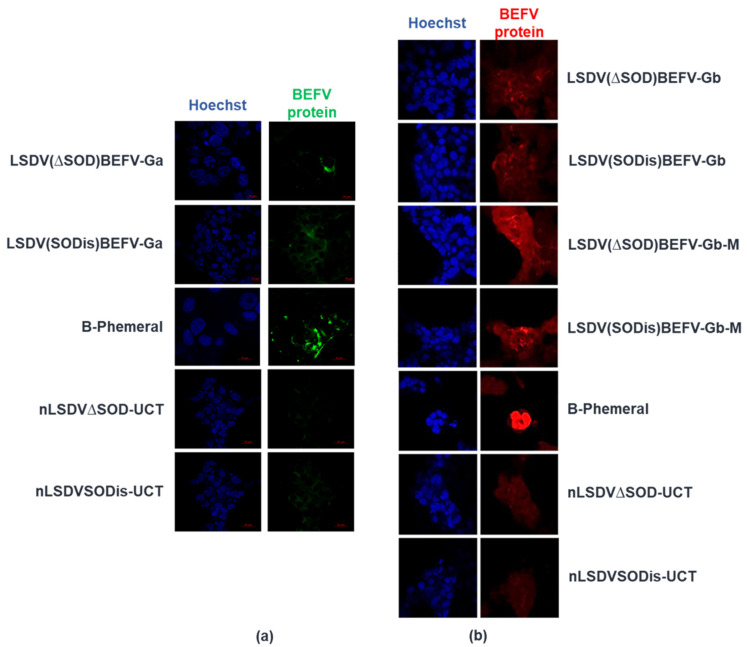
BEFV protein expression detected by immunofluorescence. MDBK cells grown on chamber slides were infected with B-Phemeral (positive control) and each of the vaccines at an MOI of 0.1 for 48 h. The two parent LSDV vaccines (nLSDV∆SOD-UCT and nLSDVSODis-UCT) were used as negative controls. Anti-B-Phemeral rabbit serum was used as the primary antibody for all samples (1:1000 dilution). (**a**) detection of BEFV Ga expression (green) using donkey anti-rabbit Alexa488 secondary antibody (1:500); (**b**) detection of BEFV Gb (red) and BEFV-Gb-M (red) using anti-rabbit CY3 secondary antibody (1:500). Nucleic acid was stained with Hoechst (blue).

Two variants of the Neethling vaccine strain of LSDV, nLSDV∆SOD-UCT and nLSDVSODis-UCT [29], were used as parent viruses in the construction of candidate dual vaccines against LSD and BEF. Figure 2 shows, diagrammatically, the design of the LSDV recombinants, whereby the foreign gene cassette was inserted between LSDV open reading frames (ORFs) 49 and 50, which are highly conserved and transcriptionally convergent [41]. The two recombinants, LSDV(∆SOD)BEFV-Ga and LSDV(SODis)BEFV-Ga, were made to express the BEFV G protein gene derived from an Australian BEFV sequence (Ga) together with the red fluorescent protein, mCherry, as a marker. LSDV(∆SOD)BEFV-Gb and LSDV(SODis)BEFV-Gb expressed a BEFV G protein gene derived from a consensus South African sequence (Gb) as well as the green fluorescent protein (eGFP) as a marker. LSDV(∆SOD)BEFV-Gb-M and LSDV(SODis)BEFV-Gb-M expressed the matrix (M) protein together with the South African Gb protein and eGFP. All G protein genes were expressed from the vaccinia virus mH5 promoter (PmH5) and the M gene was expressed from a modified fowlpoxvirus promoter (PmFPV). The six recombinants were confirmed to be correct by PCR (Figure 3) and Sanger sequencing of the gene cassette inserted between LSDV ORFs 49 and 50. BEFV gene expression was verified by immunofluorescence (Figure 4).

### 3.2. Neutralizing Antibody Responses Elicited by LSDV-BEFV Vaccine Candidates in a Rabbit Model

The candidate vaccines were initially tested in a small animal model (rabbit) to determine which BEFV immunogens and LSDV vaccine backbones to take forward into cattle, a permissive host for LSDV.

#### 3.2.1. Comparison of Different BEFV Gene Inserts

**Figure 5:**
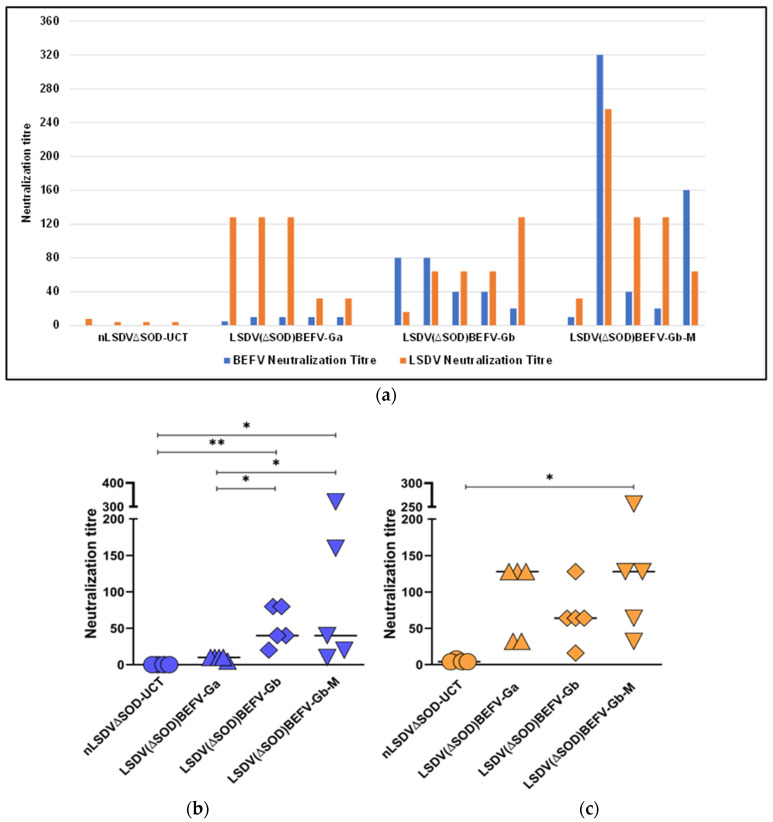
Comparison of dual vaccines expressing different BEFV gene inserts in a rabbit model. Rabbits were divided into groups of 5, and each animal was inoculated intramuscularly with 10^6^ ffu of the respective vaccines LSDV(∆SOD)BEFV-Ga, LSDV(∆SOD)BEFV-Gb and LSDV(∆SOD)BEFV-Gb-M. nLSDV∆SOD-UCT was given to a group of 4 animals as a negative control for BEFV. Rabbits were given three doses of homologous vaccine at 28-day intervals and neutralization was tested on serum taken 14 days post final inoculation. Neutralization titres are expressed as the reciprocal of the dilution required to neutralize virus in 50% or more of wells of cells infected with BEFV or LSDV, respectively. (**a**) graph showing individual animal responses to both BEFV (blue) and LSDV (orange). BEFV (blue) and LSDV (orange) responses to the dual vaccines, expressing different BEFV inserts, were compared in (**b**,**c**) respectively. Statistical analysis was conducted using the Kruskal–Wallis test with Dunn’s multiple comparison test. Horizontal lines indicate median values. * *p* < 0.05, ** *p* < 0.01.

The same LSDV vector backbone was used to compare the three different BEFV gene inserts. As a control, one group of rabbits (*n* = 4) was inoculated with the vector backbone nLSDV∆SOD-UCT. Five groups of rabbits (*n* = 5) were vaccinated with LSDV(∆SOD)BEFV-Ga, LSDV(SODis)BEFV-Ga, LSDV(∆SOD)BEFV-Gb, LSDV(SODis)BEFV-Gb, or LSDV(∆SOD)BEFV-Gb-M. After three vaccinations, all animals elicited positive neutralizing responses to LSDV (titres of 1/4 to 1/256) and BEFV (titres of 1/5 to 1/320). Figure 5a,b shows the BEFV neutralization titres to be higher (1/20 to 1/80) for rabbits vaccinated with LSDV(∆SOD)BEFV-Gb (African consensus BEFV G protein sequence) than rabbits vaccinated with LSDV(∆SOD)BEFV-Ga (Australian BEFV G protein sequence) (1/5 to 1/10). The BEFV neutralization titres of rabbits vaccinated with LSDV(∆SOD)BEFV-Gb-M were variable, ranging from 1/10 to 1/320. There was no statistical significant difference between LSDV(∆SOD)BEFV-Gb and LSDV(∆SOD)BEFV-Gb-M. Interestingly, the LSDV-BEFV recombinants all elicited higher LSDV neutralization titres than the LSDV backbone alone (Figure 5a). A statistically significant difference in LSDV response was observed between nLSDV∆SOD-UCT and LSDV(∆SOD)BEFV-Gb-M (Figure 5c). LSDV(∆SOD)BEFV-Gb-M was chosen as the vaccine of choice to use in a comparison of the two LSDV backbones nLSDV∆SOD-UCT and nLSDVSODis-UCT.

#### 3.2.2. Comparison of Different LSDV Backbones with the Same BEFV (Gb-M) Gene Inserts

**Figure 6:**
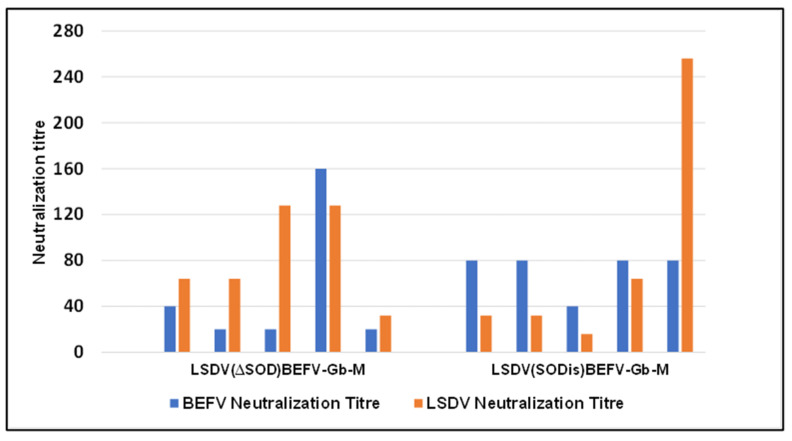
Comparison of two different LSDV vector backbones with the same BEFV gene inserts. Rabbits (*n* = 5 per group) were given three inoculations, 28 days apart, of either LSDV(∆SOD)BEFV-Gb-M or LSDV(SODis)BEFV-Gb-M, intramuscularly, at a dose of 10^6^ ffu per rabbit. Blood was taken 14 days after the final inoculation and serum was tested for neutralization of BEFV and LSDV. Neutralization titres are expressed as the reciprocal of the dilution required to neutralize virus in 50% or more of wells of cells infected with BEFV (blue) or LSDV (orange), respectively.

A second experiment was performed in rabbits, following the same procedure, to compare the two different LSDV vector backbones with the BEFV Gb and M gene inserts (Figure 6). All rabbits, inoculated with either LSDV(∆SOD)BEFV-Gb-M or LSDV(SODis)BEFV-Gb-M, elicited positive neutralizing responses to both LSDV and BEFV, but no difference could be observed between the two groups (*p* = 0.1825 for the BEFV neutralization titres and *p* = 0.318 for the LSDV neutralization titres; Mann–Whitney test). Because rabbits are non-permissive to LSDV growth, it was hypothesized that a stronger response would be elicited in a bovine host, and a difference in the host response to the LSDV vector backbone may be observed, due to the presence or absence of the SOD gene homologue.

### 3.3. Testing of LSDV(∆SOD)BEFV-Gb-M and LSDV(SODis)BEFV-Gb-M in Cattle

Two groups (*n* = 10) of cattle were vaccinated twice, four weeks apart, with homologous LSDV(∆SOD)BEFV-Gb-M or LSDV(SODis)BEFV-Gb-M vaccines and monitored daily for clinical signs of disease. Neither of the groups of cattle showed clinical signs of LSD, other than a raised temperature on day 1 post vaccination, which returned to normal the next day. There was no rise in temperature following the boost vaccination, given 32 days post initial vaccination. During the course of the experiment, two animals (ID 109 and 128), in the LSDV(∆SOD)BEFV-Gb-M group, died prior to the boost vaccination and another four (ID 120, 121, 104 and 116), two from each group, died prior to the challenge at day 201. All deaths were unrelated to the vaccinations. Three animals died as a result of acute, haemorrhagic fibrinonecrotic pneumonia, probably caused by environmental stress. These were animals 128 (died on day 19 post vaccination), 104 (died on day 59 post vaccination) and 121 (died on day 117 post vaccination). Two animals succumbed to complications as a result of bloat. These were animals 109 (died on day 35 post vaccination) and 116 (died on day 200 post vaccination). Both animals had recurrent bloat and had been treated on several occasions before they died.

#### 3.3.1. Neutralization Responses to BEFV and LSDV

Table 2 shows the neutralizing responses elicited at different time points post vaccination, post booster vaccination and post LSDV challenge. After one inoculation, only 4/20 and 7/20 animals developed neutralizing antibodies against BEFV and LSDV, respectively. Following the boost vaccination, all animals developed neutralizing responses to both BEFV and LSDV. Neutralizing antibodies against BEFV persisted for >6 months post boost in all vaccinated animals. All animals vaccinated with LSDV(∆SOD)BEFV-Gb-M lost detectable LSDV neutralizing antibodies by day 201 (169 dpb), but 7/8 of the animals vaccinated with LSDV(SODis)BEFV-Gb-M retained LSDV neutralizing antibodies until the time of challenge (169 dpb).

**Figure 7:**
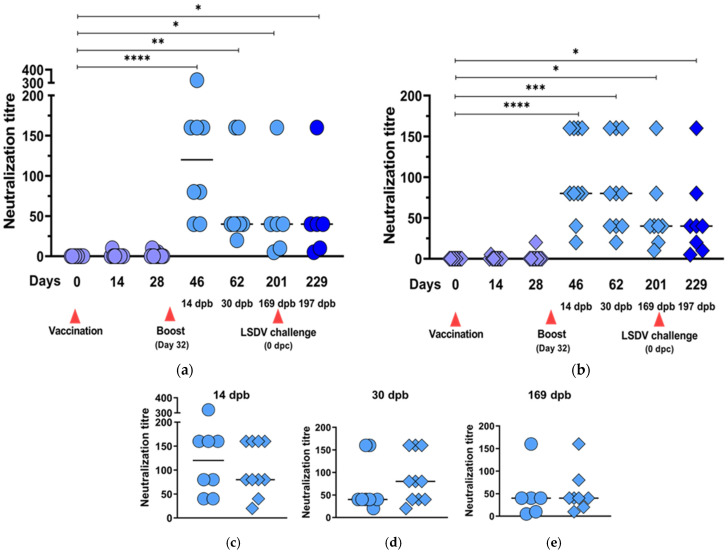
BEFV neutralization responses elicited by LSDV(∆SOD)BEFV-Gb-M (circles) and LSDV(SODis)BEFV-Gb-M (diamonds) in cattle. Two groups of cattle (*n* = 10) were inoculated subcutaneously with 10^5^ TCID_50_ per animal and boosted with 5 × 10^4^ TCID_50_ 32 days later. The animals were bled at two-week intervals and the serum tested for neutralizing responses to BEFV. Scatter plots show neutralization responses to LSDV(∆SOD)BEFV-Gb-M (**a**) and LSDV(SODis)BEFV-Gb-M (**b**) over time; different colour shades indicate responses after one (lilac) or two (light blue) vaccinations or LSDV challenge (royal blue). (**c**–**e**) show comparative BEFV responses to LSDV(∆SOD)BEFV-Gb-M (circles) and LSDV(SODis)BEFV-Gb-M (diamonds) at 14, 30 and 169 dpb, respectively. dpb = days post boost. Statistical analyses were conducted using the Kruskal–Wallis test with Dunn’s multiple comparison test (**a**,**b**) and Mann–Whitney *U* test (**c**–**e**). Horizontal lines indicate median values. * *p* < 0.05, ** *p* < 0.01, *** *p* < 0.001, **** *p* < 0.0001.

**Figure 8:**
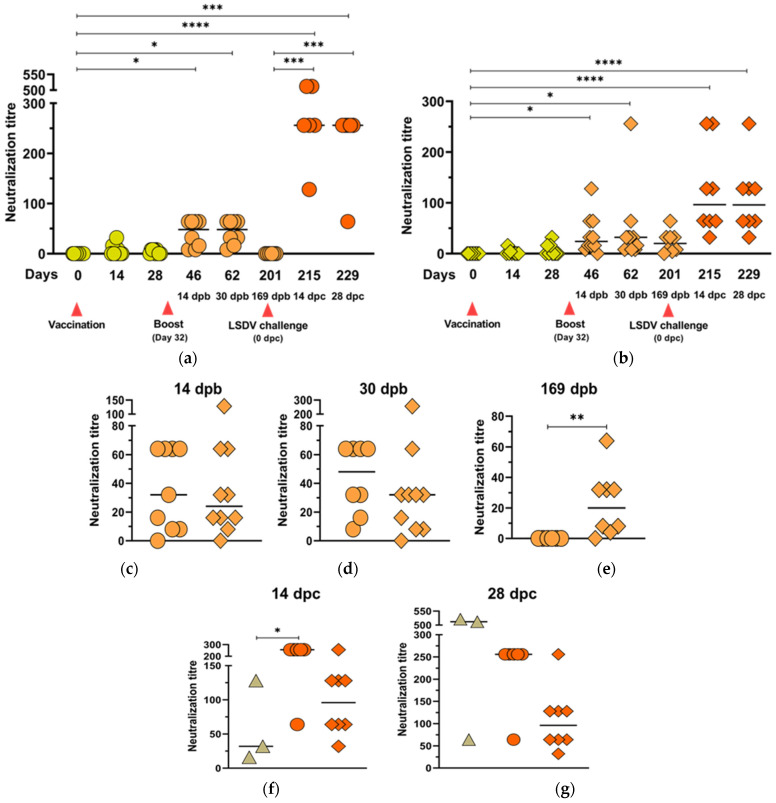
LSDV neutralization responses elicited by LSDV(∆SOD)BEFV-Gb-M (circles) and LSDV(SODis)BEFV-Gb-M (diamonds) in cattle. Two groups of cattle (*n* = 10) were inoculated with 10^5^ TCID_50_ per animal, subcutaneously, and boosted with 5 × 10^4^ TCID_50_ 32 days later. The animals were challenged with 10^7^ TCID_50_ virulent LSDV 169 days post boost. Scatter plots show LSDV neutralization responses to LSDV(∆SOD)BEFV-Gb-M (**a**) and LSDV(SODis)BEFV-Gb-M (**b**) over time; (**c**–**e**) show comparative LSDV responses to LSDV(∆SOD)BEFV-Gb-M (circles) and LSDV(SODis)BEFV-Gb-M (diamonds) at 14, 30 and 169 dpb, respectively; (**f**,**g**) show LSDV responses to the naive control animals (triangles), LSDV(∆SOD)BEFV-Gb-M (circles) and LSDV(SODis)BEFV-Gb-M (diamonds) groups, 14 and 28 dpc, respectively. Different colour shades indicate responses after one (lime) or two (orange) vaccinations or LSDV challenge (red). dpb = days post boost, dpc = days post challenge. Statistical analyses were conducted using the Kruskal–Wallis test with Dunn’s multiple comparison test (**a**,**b**,**f**,**g**) and Mann–Whitney U test (**c**,**d**,**e**). Horizontal lines indicate median values. * *p* < 0.05, ** *p* < 0.01, *** *p* < 0.001, **** *p* < 0.0001.

The increases in neutralization responses, against both BEFV and LSDV, were significantly higher for both groups of animals after the boost vaccination (Figure 7a,b and Figure 8a,b, respectively). The differences between these responses in the two groups of animals were not statistically significant (Figure 7c,d). All vaccinated animals retained BEFV neutralization antibodies up until 169 dpb (Figure 7a,b) with no significant difference between the two groups at 169 dpb (Figure 7e).

Although LSDV neutralization titres did not differ significantly between the two groups at 14 and 30 dpb (Figure 8a–d), there was a notable difference at day 201, when the group vaccinated with LSDV(∆SOD)BEFV-Gb-M showed no detectable neutralization response, whereas 7/8 of the animals from the group vaccinated with LSDV(SODis)BEFV-Gb-M had positive LSDV neutralization responses (Figure 8e). Following challenge, all animals mounted rapid and strong LSDV neutralization responses by 14 dpc, which remained at the same level at 28 dpc (Table 2 and Figure 8a,b,f,g). In comparison, the three naïve control animals developed increases in neutralizing antibody titres from 14 to 28 dpc (Table 2 and Figure 8f,g).

#### 3.3.2. Binding Antibody Responses to BEFV

**Figure 9:**
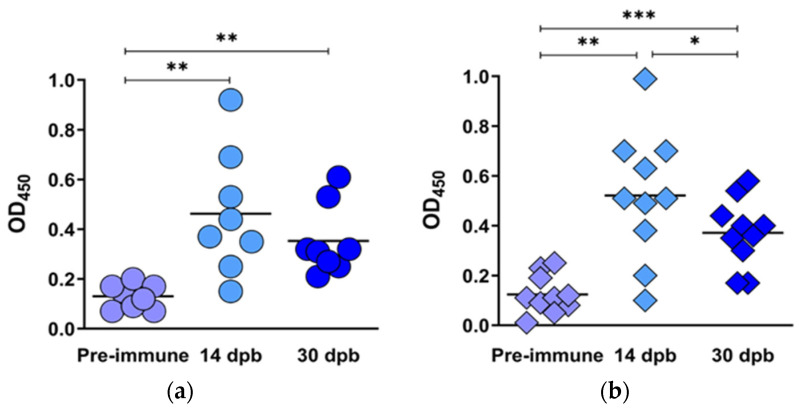
BEFV binding antibody responses elicited by LSDV(∆SOD)BEFV-Gb-M (circles) and LSDV(SODis)BEFV-Gb-M (diamonds) in cattle. Sera from the two groups of cattle were tested using a commercial ELISA kit (Unibiotest, Wuhan, China), for binding antibodies to BEFV G protein monomer of Asian lineage. Scatter plots show binding antibody responses to BEFV measured at 450 nm (OD_450_) in cattle vaccinated with LSDV(∆SOD)BEFV-Gb-M (**a**) and LSDV(SODis)BEFV-Gb-M (**b**) at pre-vaccination (lilac), 14 dpb (light blue) and 30 dpb (royal blue). Statistical analyses were conducted using repeated measures one-way ANOVA with Tukey’s multiple comparison test. Horizontal lines indicate median values. * *p* < 0.05, ** *p* < 0.01, *** *p* < 0.001.

Sera, from time points 14 and 30 dpb, were tested for BEFV binding antibodies, using pre-immune sera as a negative control (OD_450_ < 0.2) and a kit positive control (OD_450_ > 0.3). The ELISA plates were coated with a BEFV G protein monomer of Asian origin, which included the conserved linear epitope 1. All animals produced binding antibodies at 14 and 30 dpb (Figure 9a,b), with no significant differences between the groups vaccinated with LSDV(∆SOD)BEFV-Gb-M and LSDV(SODis)BEFV-Gb-M.

#### 3.3.3. Protection of Vaccinated Cattle from LSDV Challenge

**Figure 10:**
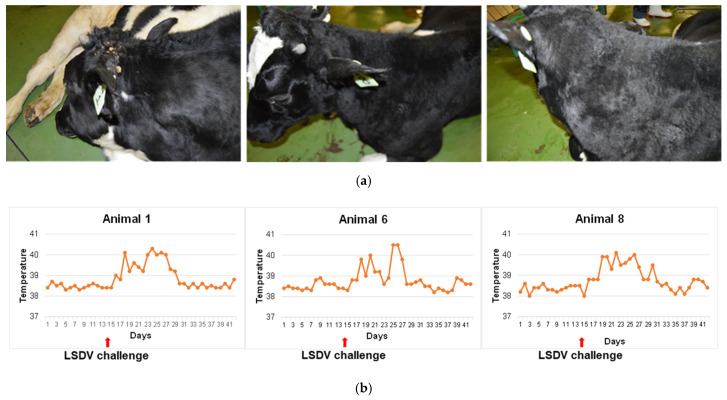
Naïve control group of cattle challenged with virulent LSDV. Three LSDV-negative cattle were infected with 10^7^ TCID_50_ virulent LSDV and observed for 28 days thereafter. (**a**) Localized swelling photographed 14 days post infection; (**b**) temperatures of the three control animals for a period of 14 days prior to challenge and 28 days post challenge.

The cattle vaccinated with LSDV(∆SOD)BEFV-Gb-M and LSDV(SODis)BEFV-Gb-M were challenged with a dose of 10^7^ TCID_50_ virulent LSDV at day 201 (almost 6 months post boost). A control group of three naïve animals was also challenged in the same way. All control animals developed localized swelling at the site of injection at 4 dpc and a raised temperature for over a week post infection. Figure 10a shows the control animals at 14 dpc and Figure 10b shows their rectal temperatures taken over a period of 6 weeks (two weeks prior to challenge and 4 weeks post challenge). In addition, the control group of animals became sensitive to touch from day 4 post infection and this lasted for approximately 10 days. Both groups of vaccinated animals developed a fever one day post challenge (data not shown), but temperatures subsided after one day. No other signs of illness were presented, showing that the vaccinated animals were protected against LSD.

#### 3.3.4. T Cell Responses to BEFV and LSDV Post LSDV Challenge

Cattle challenged with virulent LSDV were tested for CD4^+^ and CD8^+^ T cell responses prior to challenge (0 dpc) and, at weekly intervals, post LSDV challenge (7, 14 and 21 dpc). Whole blood was stimulated with either LSDV or BEFV.

##### T Cell Responses to BEFV Post Challenge

**Figure 11:**
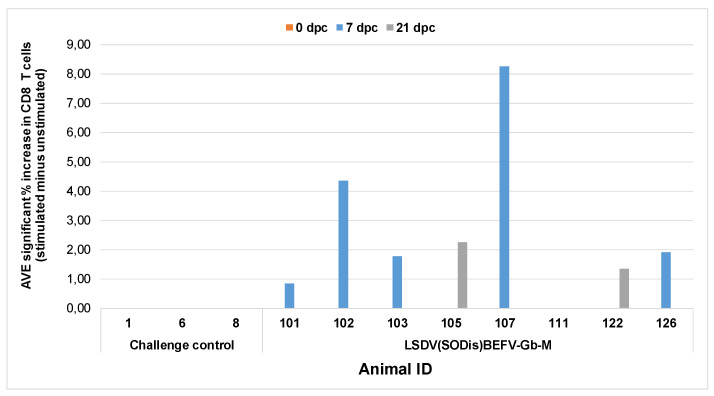
CD8^+^ T cell responses to BEFV following virulent LSDV challenge. Whole blood was stimulated with BEFV for 24 h and CD8^+^ T cells were measured. Only significant average % CD8^+^ T cells compared to control stimulants are shown. Animals 1, 6 and 8 were naïve control animals; animals 101, 102, 103, 105, 107, 111, 122 and 126 were all vaccinated with LSDV(SODis)BEFV-Gb-M. None of the animals vaccinated with LSDV(∆SOD)BEFV-Gb-M gave positive responses and there were no positive responses at 0 dpc. dpc = days post challenge.

Vaccinated cattle challenged with virulent LSDV did not develop CD4^+^ T cell responses to BEFV (data not shown). However, a significant CD8^+^ T cell response, as compared to control stimulants, was detected at 7 dpc in 5/8 animals and in two animals at 21 dpc (animal ID 105 and 122) tested in the group vaccinated with LSDV(SODis)BEFV-Gb-M (Figure 11), but not in the group vaccinated with LSDV(∆SOD)BEFV-Gb-M. The variable responses between individual animals per group and at time points are expected for outbred animals. These cells also produced low levels of IFN-γ although at a percentage increase of less than one. This indicates that a CD8 T cell response was activated by vaccination with the LSDV(SODis)BEFV-Gb-M vaccine that is specific to BEFV antigens.

##### T Cell Responses to LSDV Post Challenge

**Figure 12:**
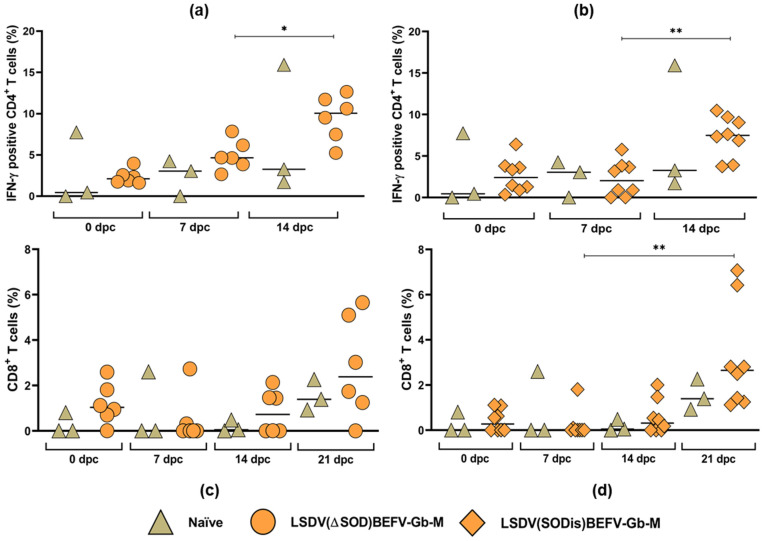
T cell responses to LSDV following virulent LSDV challenge. Whole blood was stimulated with LSDV for 24 h and IFNγ-secreting CD4^+^ and CD8^+^ T cells were measured. (**a**,**b**) the average % IFNγ- secreting CD4^+^ T cells from 0–14 dpc from cattle vaccinated with LSDV(∆SOD)BEFV-Gb-M (circles) and LSDV(SODis)BEFV-Gb-M (diamonds), respectively; (**c**,**d**) the average % CD8^+^ T cells from 0–21 dpc from cattle vaccinated with LSDV(∆SOD)BEFV-Gb-M (circles) and LSDV(SODis)BEFV-Gb-M (diamonds), respectively; the green diamonds represent unvaccinated naïve cattle. Statistical analyses were conducted using Welch one-way ANOVA with Dunnett’s T3 multiple comparison test (**a**,**b**) and Kruskal–Wallis test with Dunn’s multiple comparison test (**c**,**d**). Horizonal lines indicate median values. * *p* < 0.05, ** *p* < 0.01.

IFN-γ^+^CD4^+^ T cells, stimulated by LSDV, were produced by 14 dpc in all animals (Figure 12a,b). Most animals showed increased levels of IFN-γ^+^CD4^+^ T cells compared to the challenge control 0 dpc but, due to one outlier in the challenge control, were not significant (Figure 12a,b). There was no significant difference between the responses elicited by the three different groups, namely, naïve control animals, LSDV(∆SOD)BEFV-Gb-M- and LSDV(SODis)BEFV-Gb-M)-vaccinated animals.

A cellular immune response to LSDV mediated by CD8^+^ T cells was predominantly detected in PBMCs at 21 dpc in 5/6 animals tested in the group vaccinated with the LSDV(∆SOD)BEFV-Gb-M vaccine, in 8/8 animals in the group vaccinated with the LSDV(SODis)BEFV-Gb-M vaccine and 2/3 animals in the challenge control group (Figure 12c,d). These CD8^+^ T cells also produced low levels of IFN-γ.

Collectively, this indicates that both CD4^+^ and CD8^+^ T cell responses were activated against LSDV after challenge.

## 4. Discussion

Despite the availability of vaccines against BEF and LSD, these two diseases remain a threat to the cattle industry. Several different platforms have been used to make BEFV vaccines [10], with inactivated and live attenuated vaccines being the most widely used. Inactivated BEFV vaccines require multiple doses and, even then, are not fully protective [42,43]. A combination of live attenuated followed by killed vaccine [44] is used in Japan [11], the vaccines being based on local strains of BEFV. The live attenuated B-Phemeral, derived from an African BEFV isolate, is used in South Africa in a two-dose regimen [12].

The G protein is recognized as being antigenic [11,13,14,15,16] and has been used as a protein vaccine [13]. Secreted forms of the BEFV G protein, lacking the transmembrane portion of the protein, are being investigated as subunit vaccines [45]. Virus vectors, such as rabies [46], Newcastle Disease virus [40], vaccinia virus [14] and LSDV [47], which express the BEFV G protein, continue to be explored as platforms for BEFV vaccine development. Proof of concept that a recombinant poxvirus could be used to protect cattle is given by Hertig et al., (1996) who demonstrated that the vaccinia virus expressing BEFV G protein could protect cattle from BEFV challenge [14]. Since then, LSDV has been explored as a vector for novel BEFV vaccines [47,48]. All previous research on poxvirus recombinants expressing BEFV proteins is based on the Australian BEFV G protein [14,47,48]. The LSDV-BEFV recombinants were made many years ago, using the LSDV Neethling vaccine as a backbone, and included the metabolic selection gene, guanine phosphoribosyl transferase, which is not present in the recombinants described in this paper. In addition, the thymidine kinase [47] or ribonucleotide reductase [48] genes were disrupted for insertion of the foreign gene cassette. Disruption of these genes further attenuates LSDV, which is not desirable. These previous studies on LSDV-BEFV recombinants could not demonstrate protection from BEFV challenge. In our study, an intergenic site, located between two highly conserved open reading frames, LSDV ORFs 49 and 50, was used. This site was chosen as a stable site of insertion, which would not cause any gene disruption in LSDV. No antibiotic resistance genes were incorporated into the recombinants.

Recent evidence has shown that the G protein sequences of African BEFV isolates differ from those from other parts of the world [12,18] and sequence alignment has revealed differences in amino acid sequences in the known antigenic epitopes of the G protein between the African isolates and those from other parts of the world [12]. However, the linear G1 epitope has only one conserved amino acid difference (I496V), suggesting it may confer some cross-protection. Rabbits vaccinated with LSDV(∆SOD)BEFV-Ga, which expresses an Australian G protein, developed low titres (1/5 to 1/10) of neutralizing antibodies against B-Phemeral. The cross neutralization could be attributed to the conservation of the G1 epitope. An ELISA test, based on a subunit Asian G protein containing the G1 epitope, was used to test vaccinated cattle sera for binding antibodies to this BEFV subunit G protein. All animals, which were vaccinated with vaccines expressing the South African G protein, elicited antibodies which bound to the Asian G protein (Figure 9). These results are not surprising as antigenic cross-reactivity has been shown between isolates from Australia, China, Japan, Kenya, Nigeria and South Africa [11].

Rabbits vaccinated with recombinant LSDV expressing the South African consensus G protein, LSDV(∆SOD)BEFV-Gb, elicited a significantly increased BEFV neutralization response compared to those vaccinated with LSDV(∆SOD)BEFV-Ga (Figure 5b). In contrast, there was no significant difference in LSDV neutralization titres between these two groups (Figure 5c), and the LSDV(∆SOD)BEFV-Ga group tended towards a slightly higher LSDV neutralization response. The stronger BEFV neutralization responses are most likely due to the identity of all four antigenic epitopes conserved amongst the African isolates. The strong BEFV neutralizing responses induced by Gb confirmed that the design of the South Africa consensus G protein was appropriate.

The highest neutralization titres were generated by rabbits vaccinated with LSDV(∆SOD)BEFV-Gb-M; however, there was no statistical significance between the groups to definitively conclude that the M protein gene improved the response. Whether the M protein aids the formation of virus-like particles or not is still to be determined. The two candidate vaccines LSDV(∆SOD)BEFV-Gb-M and LSDV(SODis)BEFV-Gb-M were compared to determine whether the absence or presence, respectively, of the LSDV SOD-homologue gene would influence immunogenicity. No difference was found between the two groups of rabbits immunized with the different vaccines. The strong neutralization responses elicited against both BEFV and LSDV in a non-permissive (rabbit) host justified testing the vaccine candidates in cattle, the natural host for LSDV. The two vaccines, LSDV(∆SOD)BEFV-Gb-M and LSDV(SODis)BEFV-Gb-M, expressing both Gb and M, were therefore tested in cattle.

The permissive bovine animal model was thought to be more sensitive to the effects of the LSDV SOD-homologue on immunogenicity and clinical response to the vaccine. An earlier pilot experiment to compare nLSDV∆SOD-UCT and nLSDVSODis-UCT in the same animal suggested that there could be a difference with respect to pathology caused by the two different LSDV backbones [29], but no differences were observed between the two vaccines LSDV(∆SOD)BEFV-Gb-M and LSDV(SODis)BEFV-Gb-M in this study. Lesions were produced at the sites of inoculation in 1/10 and 3/10 animals vaccinated with LSDV(∆SOD)BEFV-Gb-M and LSDV(SODis)BEFV-Gb-M, respectively, and these resembled the lesions regularly observed in cattle vaccinated with the Neethling strain of LSDV.

A drawback of this study was the inability to test the vaccines in a virulent BEFV challenge experiment, due to the unavailability of virulent BEFV challenge virus. The preparation of virulent BEFV challenge virus has been a major hurdle in testing BEFV vaccines for other groups too [14,40,47]. However, we were able to demonstrate that the two vaccines, LSDV(∆SOD)BEFV-Gb-M and LSDV(SODis)BEFV-Gb-M, elicited neutralizing antibodies to BEFV after two inoculations given four weeks apart, with titres ranging from 1/20 to 1/320 at 14 dpb. The BEFV neutralization responses were durable for at least six months in all vaccinated animals. This is encouraging as BEFV neutralizing antibodies have been shown to wane after four months post vaccination with inactivated BEFV [44]; and a neutralization titre of 1/5 or 1/6 has been associated with protection against BEFV infection [49]. The BEFV neutralization titres ranged from 1/5 to 1/160 at day 229 (197 dpb) in this study (Table 1).

Although all animals, vaccinated with either LSDV(∆SOD)BEFV-Gb-M or LSDV(SODis)BEFV-Gb-M, developed strong antibody responses to BEFV, the group vaccinated with LSDV(SODis)BEFV-Gb-M, and not that vaccinated with LSDV(∆SOD)BEFV-Gb-M, gave an increased CD8^+^ T cell response at seven days post virulent LSDV challenge. The BEFV memory cells induced by vaccination will reside in secondary lymph organs. These CD8^+^ T cells are likely in circulation in PBMCs post challenge for immune surveillance induced by the innate detection of a viral infection after the LSDV challenge. Since there was no BEFV detected in the host by these cells, clonal expansion of BEFV specific CD8^+^ T cells did not occur in vivo and thus were only detected in PBMC in vitro for a limited time.

This study, using the two LSDV vaccine backbones, nLSDV∆SOD-UCT and nLSDVSODis-UCT [29], showed both vaccines to be safe and immunogenic, eliciting strong humoral (neutralizing antibody) and T cell responses to LSDV, as well as to provide protection (100%) against virulent LSDV challenge. All animals in the naïve control group developed clinical symptoms of LSD, indicating that the protection was due to vaccination.

The duration of LSDV neutralizing antibodies differed according to the vaccine backbone. Although all animals in both groups were protected against challenge, the group vaccinated with LSDV(SODis)BEFV-Gb-M showed a more durable LSDV neutralizing responses compared to the group vaccinated with LSDV(∆SOD)BEFV-Gb-M. All animals developed LSDV neutralizing antibodies by 30 dpb, with SNT titres ranging from 1/8 to 1/256. At day 201 (169 days post boost), all animals vaccinated with LSDV(∆SOD)BEFV-Gb-M had dropped LSDV neutralizing antibody titre to undetectable levels, whereas 7/8 animals vaccinated with LSDV(SODis)BEFV-Gb-M had positive neutralizing antibody titres (1/4 to 1/64). Interestingly, post challenge, the LSDV(∆SOD)BEFV-Gb-M group developed higher neutralizing antibody responses than the LSDV(SODis)BEFV-Gb-M by 14 days post challenge. A possible explanation for this could be that the challenge virus replicated in the animals with undetectable neutralizing antibodies and the higher viral load stimulated a higher response needed to control the challenge. The neutralization titres remained constant until the end of the experiment at 28 dpc. In comparison, the naïve control group of animals showed a slower increase in neutralization titres, with higher titres at 28 dpc compared to 14 dpc. This confirms the memory response in previously vaccinated animals compared to the naïve animals.

The T cell responses to LSDV were predominantly CD4^+^ in nature, which developed by 14 dpc. The CD8^+^ T cell responses to LSDV were lower than the CD4+ responses and developed later, at 21 dpc (Figure 12). The CD4^+^ cells were the main producers of IFN-γ. The more delayed response of the LSDV specific CD8^+^ T cell kinetics compared to that of BEFV is likely due to homing of the cells to the site of infection after challenge and detection in circulating PBMCs once the virus at the challenge site was cleared.

An interesting observation with repeated LSDV-BEFV inoculations was the ability to boost antibody responses to both BEFV and LSDV. This was also observed in our previous study on a LSDV-rabies recombinant [50], and LSDV re-vaccination of cattle has been shown to significantly increase antibody titre to LSDV [51]. One of the concerns with viral vectored vaccines is neutralization of the vector, preventing use of the same vector for boosting responses to the transgene product [52]. However, multiple immunizations with LSDV did not result in prevention of infection with the boosting virus. The challenge experiment would indicate that neutralizing responses played a role in controlling infection together with T cell responses. This is an indication that LSDV could be used to deliver multivalent vaccines, or alternatively, different vaccines at different times without the immunity to LSDV preventing infection with the recombinant virus.

The recombinant vaccines described in this paper are potential candidates for dual vaccines against LSD and BEF. The two recombinants LSDV(∆SOD)BEFV-Gb-M and LSDV(SODis)BEFV-Gb-M warrant further testing in the field as they were both safe at the dosage tested, which was 10 times higher than the standard LSDV vaccine dose. The cattle used in this experiment were Friesian dairy cattle, which were expected to be the most sensitive cattle to LSDV vaccines. No evidence of Neethling associated disease was observed. However, the vaccines need to be tested in different species of cattle to further determine dosage, immunogenicity and safety. We hypothesize that the recombinants expressing the BEFV Ga protein would be more suited to use in Asia and Australia; and those vaccines expressing BEFV Gb would be more suitable for use in Africa.

## 5. Conclusions

Six candidate vaccines have been developed to simultaneously immunize cattle against BEF and LSD. In a rabbit model, all vaccines elicited neutralizing responses to the live attenuated B-Phemeral vaccine strain of BEFV, including the vaccine candidates expressing the Australian BEFV Ga gene. With Africa in mind, greater focus was on those vaccines based on the consensus African sequence of BEFV (Gb) as vaccines expressing the Gb protein are likely to be more protective in the African setting. It could not conclusively be shown that the BEFV M gene improved immunogenicity, but the highest neutralization titres were produced by rabbits vaccinated with LSDV expressing both Gb and M protein genes. The two candidate vaccines, LSDV(∆SOD)BEFV-Gb-M and LSDV(SODis)BEFV-Gb-M, elicited neutralizing responses to both BEFV and LSDV in cattle. In addition, they conferred protection against virulent LSDV challenge, inducing rapid, strong neutralizing responses post challenge as well as CD4^+^ and CD8^+^ T cell responses.

## 6. Patents

A provisional patent application has been filed for the vaccine LSDV(SODis)BEFV-Gb-M, application number: SPOORSA-sa_cases.0153757.PA176615/P.

## Figures and Tables

**Table 1:** Summary of components used in the construction of six different LSDV-BEFV dual vaccines.

Vaccine	LSDV Parent Virus	Genes Inserted between LSDV ORFS 49 and 50 *	Marker *
nLSDV∆SOD-UCT	Neethling LSDV vaccine with SOD homolog gene (ORF 131) deleted [29]	parent virus with no gene insertions	None
nLSDVSODis-UCT	Neethling LSDV vaccine with full length modified SOD homolog gene (ORF 131) [29]	parent virus with no gene insertions	None
LSDV(∆SOD)BEFV-Ga	nLSDV∆SOD-UCT	BEFV glycoprotein gene of Australian origin (Ga) [14]	mCherry
LSDV(SODis)BEFV-Ga	nLSDVSODis-UCT		
LSDV(∆SOD)BEFV-Gb	nLSDV∆SOD-UCT	BEFV glycoprotein gene based on consensus sequence of South African G protein genes (Gb) [18]	eGFP
LSDV(SODis)BEFV-Gb	nLSDVSODis-UCT		
LSDV(∆SOD)BEFV-Gb-M	nLSDV∆SOD-UCT	BEFV glycoprotein gene based on consensus sequence of South African G protein genes (Gb) [18] and matrix gene (M) [19]	eGFP
LSDV(SODis)BEFV-Gb-M	nLSDVSODis-UCT		

* Both BEFV glycoprotein genes (Ga [14] and Gb [18]) are under the control of the vaccinia virus mH5 promoter [33], the BEFV matrix [19] and mCherry genes are controlled by the modified fowlpoxvirus (mFPV) promoter [34], and the eGFP gene is controlled by the synthetic vaccinia virus promoter (pS) [35,36].

**Table 2:** Neutralization responses of cattle to BEFV (blue) and LSDV (orange). Titres are expressed as the reciprocal of the dilution required to neutralize the virus in 50% or more of wells of cells infected with BEFV or LSDV, respectively. The higher the titre the darker the shade. Titres < 1/5 for BEFV and <1/4 for LSDV were regarded as -ve. pre-im = pre-immunisation, dpv = days post vaccination, dpb = days post boost, dpc = days post challenge; asterisks show animals which developed lesions at sites of inoculation (sizes of lesions in brackets); ☨ animal died (unrelated to vaccination).

Animal ID	BEFV Neutralization Titre							LSDV Neutralization Titre							
Day	0	14	28	46	62	201	229	0	14	28	46	62	201	215	229
	pre-im	14 dpv	28 dpv	14 dpb	30 dpb	169 dpb	197 dpb	pre-im	14 dpv	28 dpv	14 dpb	30 dpb	0 dpc	14 dpc	28 dpc
LSDV(∆SOD) BEFV-Gb-M															
110	-ve	-ve	5	320	160	160	160	-ve	-ve	-ve	8	32	-ve	256	256
112	-ve	-ve	-ve	80	20	5	5	-ve	-ve	-ve	64	64	-ve	128	64
113	-ve	-ve	5	40	40	40	40	-ve	16	8	64	64	-ve	256	256
115 * (2.5 cm)	-ve	10	10	160	40	40	40	-ve	8	8	32	32	-ve	256	256
120	-ve	-ve	-ve	160	40	☨	☨	-ve	16	8	64	64	☨	☨	☨
121	-ve	-ve	-ve	80	40	☨	☨	-ve	-ve	-ve	8	8	☨	☨	☨
123	-ve	-ve	-ve	40	40	40	40	-ve	32	8	16	16	-ve	512	256
129	-ve	-ve	-ve	160	160	10	10	-ve	-ve	-ve	64	64	-ve	512	256
109 ☨	-ve	-ve	-ve	☨	☨	☨	☨	-ve	-ve	-ve	☨	☨	☨	☨	☨
128 ☨	-ve	-ve	☨	☨	☨	☨	☨	-ve	-ve	☨	☨	☨	☨	☨	☨
LSDV(SODis) BEFV-Gb-M															
101	-ve	-ve	-ve	80	80	80	80	-ve	4	-ve	8	8	8	128	128
102	-ve	-ve	-ve	160	160	40	40	-ve	-ve	-ve	64	64	32	64	128
103 * (2.2 cm)	-ve	-ve	-ve	80	40	40	20	-ve	-ve	-ve	32	32	8	256	128
104	-ve	-ve	-ve	20	20	☨	☨	-ve	-ve	-ve	-ve	8	☨	☨	☨
105	-ve	-ve	-ve	40	40	20	10	-ve	-ve	-ve	16	16	4	64	64
107	-ve	-ve	-ve	160	80	10	5	-ve	-ve	-ve	16	32	-ve	64	32
111 * (1.8 cm)	-ve	5	20	160	160	160	160	-ve	16	32	128	256	64	128	64
116	-ve	-ve	-ve	160	160	☨	☨	-ve	-ve	-ve	16	8	☨	☨	☨
122 * (3.2 cm)	-ve	-ve	-ve	80	80	40	40	-ve	-ve	16	64	32	32	32	64
126	-ve	-ve	-ve	40	40	40	40	-ve	4	16	32	32	32	256	256
													0 dpc	14 dpc	28 dpc
Naïve Control Animals															
1 * (>6 cm)													-ve	128	512
6 * (>6 cm)													-ve	16	64
8 * (>6 cm)													-ve	32	512

## Data Availability

Not applicable.
